# Realising the full potential of MR-PHeWAS in cancer

**DOI:** 10.1038/s41416-020-01165-0

**Published:** 2020-11-25

**Authors:** Jack Bowden

**Affiliations:** Exeter Diabetes Group (ExCEED), College of Medicine and Health, University of Exeter, Exeter, UK

**Keywords:** Epidemiology, Genetics research

## Abstract

MR-PHeWAS is a powerful new design for discovering causal mechanisms between a disease and its many candidate risk factors in a hypothesis-free manner. This technique has great potential in the field of cancer research, provided that both powerful and principled statistical approaches are used.

## Main

It has long been known that our genes can influence, in a very small way, many aspects of our health and behaviour at a population level. Since they are randomly allocated and fixed at the point of conception, we can view ourselves as having been randomised into many concurrent and life-long natural experiments. Mendelian randomisation^1^ (MR) is the science of exploiting this basic premise, by augmenting the analysis of observational data with genetic information in a bid to uncover the causal mechanisms of disease.

Over the past two decades MR has increased in prominence, being widely applied across the medical and social sciences. The approach typically assumes that a carefully selected group of genetic variants, which are usually single-nucleotide polymorphisms (SNPs), satisfy the instrumental variable (IV) assumptions. That is, they are (i) robustly associated with a modifiable exposure of interest, (ii) independent of any confounders of the exposure–outcome relationship and (iii) can only influence the outcome through the exposure. Two main reasons why MR is now widely used are the range of statistical methods available that are robust to certain violations of the IV assumptions and the ever-expanding set of candidate genetic instruments available for common disease traits. One of the most studied violations, termed horizontal pleiotropy,^2^ occurs when a genetic variant affects the outcome through a separate and possibly unknown mechanism than the exposure under investigation.

In a traditional ‘one-sample’ MR study, individual level data from a single cohort is used to construct a genetically predicted exposure, or ‘polygenic risk score’, from many genetic variants. Its coefficient of association with the outcome is taken as the causal effect estimate. Alternatively, MR analyses can be conducted by combining summary estimates of SNP–trait associations from two or more genome‐wide association studies (GWAS) to produce variant-specific causal estimates, which are then meta-analysed to produce an overall causal estimate.^3^ Researchers are increasingly extending the scope of their investigations by simultaneously performing thousands of MR analyses across the phenome to uncover the traits with the strongest evidence of a causal mechanism. This technique, termed ‘MR-PheWAS’ (MR-phenome-wide association study), is used to prioritise further epidemiological studies and has also been used to prioritise potential drug targets in the pharmaceutical arena.^4^

Glioma is an aggressive cancer responsible for the vast majority of brain tumours, and the rationale for an MR investigation into its causes is strong: the prognosis for many patients has long been poor, failing to improve in line with many other cancers, with 5-year survival for glioblastoma being only 5%. Despite differences in the incidence across countries, which hints that environmental or lifestyle factors could play a role, only exposure to ionising radiation has so far been definitively linked. In this issue of the *British Journal of Cancer*, Saunders et al.^5^ use an MR-PHeWAS design in an attempt to uncover causal mechanisms for glioma and avoid the problems of unmeasured confounding and reverse causation that affect traditional epidemiological investigations. They used summary statistics on over 8000 individual variants from two separate GWAS, capturing their association with 316 intermediate phenotypes and glioma risk. In their analysis, no single phenotype was estimated to have a strong enough causal effect to fall below a pre-specified Bonferroni-adjusted 5% type I error threshold, although 13 phenotypes showed suggestive evidence of a causal association (*P* < 5%). Furthermore, when fully pleiotropy-robust MR methods were used, only telomere length, low-density lipoprotein and glycated haemoglobin remained suggestive.

Although the analysis was inconclusive, and the methodology used sound, their work highlights a number of current limitations in the statistical methods routinely applied to MR-PHeWAS.

### Pleiotropy-robust methods and power

Many of the strongest results in their analysis were driven by a single variant (e.g. the effect of telomere length on glioma risk through the *TERT* gene), but the precision of their overall causal effect was dramatically diminished due to the presence of substantial heterogeneity across the remaining SNPs. Modern MR approaches generally utilise large numbers of SNPs, but interpret heterogeneity in causal estimates across SNPs as a sign of horizontal pleiotropy.^3^ However, they still assume that the majority of the genetic signals are correct, or they are all correct ‘on average’. This may be true, but it could also be the case, for example, that *TERT* is the *only* reliable genetic instrument in the analysis. This presents a future challenge for MR approaches in being able to separate out the small kernel of truth in a larger sea of biased data. At the very least, common MR power calculators^6^ need to be updated to account for the presence of pleiotropy in inducing heterogeneity into the data, as well as the application of pleiotropy-robust (but less efficient) analyses.

### Accounting for multiplicity in MR-PHeWAS

In the MR-PHeWAS field, care needs to be taken to understand the issue of multiplicity when looking simultaneously at many potential causal hypotheses. Indeed, Saunders et al.^5^ applied a standard Bonferroni correction and found that no single analysis passed the multiplicity corrected threshold. Whilst a Bonferroni procedure is guaranteed to control the family-wise error rate (FWER), it can be unnecessarily conservative when there are a large number of tests and the test statistics are correlated. There is great potential in applying more powerful multiplicity corrections that exploit the correlation structure between the causal parameter test statistics, such as Hochberg or Dunnett procedures, whilst maintaining control of the FWER. Another extension to explore is the use of weighted multiple testing procedures, to allow the incorporation of prior knowledge about which causal mechanisms may be the most important. To realise further improvements in power, more lenient methods for controlling the overall false discovery rate, as opposed to FWER, could also be utilised.

In summary, novel methods are urgently needed in order to fully exploit the MR-PHeWAS design. This will increase the power to discover important causal pathways for cancer and other diseases, whilst still offering protection against spurious or chance findings. Of course, it must be remembered that MR is not infallible.^7^ Replication and triangulation of findings using different data sources,^8^ and if possible, benchmarking against randomised trials will also be vital going forward.

## Data Availability

Not applicable.
